# Systemic Lupus Erythematosus, Its Impact on Selected Cardiovascular Risk Factors, and Correlation with Duration of Illness: A Pilot Study

**DOI:** 10.1155/2020/7025329

**Published:** 2020-11-06

**Authors:** Brygida Przywara-Chowaniec, Dominika Blachut, Jan Harpula, Marcin Bereś, Agnieszka Nowak, Ewa Nowalany-Kozielska

**Affiliations:** ^1^2nd Department of Cardiology, Faculty of Medical Sciences in Zabrze, Medical University of Silesia, Katowice, Poland; ^2^Department of Chemistry, Faculty of Medical Sciences in Zabrze, Medical University of Silesia, Katowice, Poland

## Abstract

Systemic lupus erythematosus is a rare autoimmune disease. It leads to an increased production of proinflammatory molecules that accelerates atherogenesis and could cause an endothelium dysfunction. The aim of the study was to assess cardiovascular risk factors such as BMI and lipid profile as well as left ventricular ejection fraction among patients with SLE, and a correlation of these factors with duration of the disease. *Materials and Methods*. The researched group consisted of patients with SLE, being under control of the outpatient clinic of cardiology. This group included 38 patients among whom 34 were women (56.17 ± 11.05 years) and 4 were men (65.50 ± 9.22 years). The control group consisted of 19 healthy women (53.31 ± 11.94 years) and 2 healthy men (38.51 ± 7.53 years). Measurements were taken in the same conditions by trained medical staff. *Results*. Excessive body weight (BMI >25 kg/m^2^) was more frequent in the SLE group, but it was not statistically significant (55.26% vs. 52.38%, *p* = 0.6159). LVEF values were lower in their searched group, and this factor showed statistical significance (53.92% ± 6.46 vs. 58.67% ± 4.69, *p* = 0.0044). Thickness of the IMT was higher and statistically important among patients with SLE, both in left (1.22 ± 0.27 mm vs. 0.7 ± 0.21 mm, *p* = 0.0001) and right common carotid artery (1.16 ± 0.26 mm vs. 0.59 ± 0.15 mm, *p* = 0.0001), compared to the controls. *Conclusions*. Patients with SLE are at greater risk of developing cardiovascular diseases as the illness progresses. The activity of the disease according to the SLEDAI-2K scale may have an impact on the LVEF values which was significantly decreased in the group with active disease, but further thorough investigation is required to fully evaluate the impact of individual components of the disease and its treatment on the CVD development and mortality.

## 1. Introduction

Systemic lupus erythematosus (SLE) is a chronic autoimmune disease that is characterized by presence of the antibodies directed against different body antigens which could result in many nonspecific clinical symptoms. Women are the most often affected by this disease, whereas almost 20% of SLE cases are described as juvenile lupus, which is characterized by more severe course [1]. The heart is one of the most commonly occupied organs with a frequency of over 50% of SLE cases, and it should be noted that practically all of its structures can be attacked. Characteristic manifestations of heart lupus are in majority of cases myocarditis and pericarditis. The inflammation of pericardium is recognized by the American College of Rheumatology (ARA/ACR) guidelines as a one of the clinical criteria of the SLE [2]. The transthoracic echocardiography (TTE) is often performed when it comes to the assessment of the cardiovascular system among the SLE patients, as it is an easy to perform and effective way to assess the differences in the heart functioning and structure, thus TTE should be considered as a standard examination procedure among these patients [3]. As a result of increased production of the proinflammatory molecules, SLE accelerates atherogenesis process, which may contribute to the development of cardiovascular diseases, which are considered the main cause of death among patients with SLE [4]. Atherosclerosis is considered as a major factor of the cardiovascular disease (CVD). However, pathophysiology of this disease is complex and it is an oversimplification to assume that only atherosclerosis plays a vital role in its origin. One of the components that are relevant is a coronary microvascular dysfunction (CMD), which could lead to the myocardial infarction and to the heart failure [5].

The intima-media thickness (IMT) in common carotid arteries (CCA) is one of the parameters that gives an insight of the severity of atherosclerosis changes, and it can be measured during an ultrasonographic examination of these vessels [6]. It should be denoted that IMT values over 0.9 mm are, according to the European Society of Cardiology (ESC), a risk factors for CVD [7]. As the IMT values increase, the risk of CVD increases, as it informs as well about atherosclerosis as smooth muscle cell growth, therefore being independent CVD risk marker [8–10]. Both atherogenesis and heart occupation by the antibodies contribute to the heart failure development, which is considered a risk factor for sudden cardiac death (SCD). In order to assess the severity of the failure process, one of the routinely tested parameters is the left ventricular ejection fraction (LVEF), measured during TTE examination. The complexity of the SLE reveals itself also in the metabolic field with lipid profile abnormalities, such as hypercholesterolemia, hypertriglyceridemia, or high density and low density cholesterol irregularities, which are considered as one of the risk factors for the cardiovascular event [11]. The aim of the study was to assess cardiovascular risk factors such as IMT value ≥1.5 mm or being ≥50% greater than the segment below the enlargement, the body mass index (BMI), and a lipid profile, as well as LVEF among patients with SLE [8]. Afterwards, the aim was to calculate correlation of these factors with duration of the disease and to assess wheatear the active disease or corticosteroids treatment affect factors mentioned earlier.

## 2. Materials and Methods

The researched population consisted of patients from the 2nd Department of Cardiology, Faculty of Medical Sciences in Zabrze, Medical University of Silesia in Katowice, Poland. The study was conducted after previous acceptation by the Ethics Committee functioning at this university.

Every patient was thoroughly informed of requirements and goals of the study, after which they signed an informed consent. The study was conducted from October 2016 to January 2020, and it is still continued. 38 patients with the SLE were examined, including 34 women at age of 56.17 ± 11.05 and 4 men at age 65.50 ± 9.22 years. The control group consisted of 21 healthy adults of whom 19 were women at age 53.31 ± 11.94 year and 2 were men at 38.51 ± 7.53 years of age. The measurements were carried out in the same conditions and by qualified personnel. All examinations were performed in outpatient clinic as a part of the routine patient assessment.

Subjects included to the study were patients with SLE, both with subclinical or clinically active type of the disease. Patients with other autoimmune connective tissue disorders and with the overlap syndrome were excluded from the study, as clinical symptoms of these diseases could interfere with SLE symptoms.

SLE was diagnosed with the help of ACR criteria, and afterwards, the disease activity was assessed using Systemic Lupus Erythematosus Disease Activity Index (SLEDAI-2K). Scores equal and above 6 were an indication to recognize an active disease.

BMI was calculated according to the formula of the quotient of body mass expressed in kilogram (kg) to the square of body height (m^2^), and it was expressed in units of kg/m^2^. Each person was weighed and measured on an empty stomach. The study also included people actively smoking cigarettes.

The LVEF was calculated using modified Simpson's biplane method, during the TTE examination. The examined patient was lying on the left side of the body. Machine used to perform this test was the GE Healthcare Vivid 7 ultrasound system, with 3.6 MHz probe. The ejection fraction calculations were based on the recordings of echocardiographic images from the four- and two-chamber apical projections, each recording including at least three heart cycles. The results presented are the average of at least three LVEF measurements and are given in percent (%).

The IMT measurements were performed by the ultrasound method, using a GE Healthcare Vivid 7 ultrasound machine with a 10 MHz linear probe installed. Right and left CCA were examined while the patient was lying in supine position, with his head turned in the opposite direction. The probe was set at the longitudinal view of the vessel, and the measurements were made according to the protocol described in the American Society of Echocardiography (ASECHO) guidelines on the posterior wall of the artery, 1–2 cm below its bifurcation [12].

Blood samples for laboratory tests were obtained from each patient on an empty stomach in the morning. Lipid profile tests were performed, and parameters such as total cholesterol (TC), high-density cholesterol (HDL), low-density cholesterol (LDL), and triglycerides (TG) were measured.

Patient CVD risk score was calculated with the help of the QRISK®3 [13]. This tool was developed to estimate risk of adverse events, such as: stroke, transient ischemic attack, myocardial infarction, or angina in the next 10 years. The factors included into calculation are chronic kidney disease, migraine, corticosteroids, SLE, atypical antipsychotics, severe mental illness, erectile dysfunction, systolic blood pressure (SBP), BMI, and cholesterol/HDL ratio.

### 2.1. Statistical Analysis

To statistical analysis, the Statistica, version 13.3, software, StatSoft Inc. 2014, and the Excel Software from the Microsoft Office 2016 were used. Analysis of distribution of the tested parameters was performed using the Shapiro–Wilk test. Parameters with a normal distribution were developed using the *t*-test, while parameters not having normal distribution, using the Mann–Whitney test. To assess the correlation coefficient for parametric values, the Pearson correlation test was used, and for nonparametric values, the Spearman rank order correlation was used. The level of statistical significance was adopted at the level of *p* < 0.05.

## 3. Results

The researched group consisted of 38 people, and average age of SLE patients was 56.45 ± 11.01 years. At the time, when this study was conducted, 7 patients were active tobacco smokers. Mean duration time of the SLE was 10.21 ± 7.69 years. The analysis of the SLEDAI-2K scale showed a mean score of 6.89 ± 5.51, whereas the number of patients who scored ≥6 points was 17 (44.74%). The lipid profile analysis revealed differences between two groups, but none of them were statistically significant. The TC, LDL, and TG values were higher in the SLE group, and on the other hand, the HDL was higher among healthy controls. In the matter of the cardiovascular risk score according to the QRISK®3 calculator, the mean value among the SLE group was 15.76%.The patient's characteristics are included in Table 1.

The control group consisted of 21 patients, 19 women and 2 men, and all of whom were adults. The mean age of subjects was 51.91 ± 12.38 years, and 1 female patient was an active smoker at the time. None of them were suffering from a serious CVD of any type. Every participant signed informed consent and decided to take part in the study as a volunteer. The exclusion criteria were as follows: being diagnosed with autoimmune disease, metabolic disorder, or pregnancy. The health statuses were obtained retrospectively from previous medical records. The diabetes type 2 occurred in 2 patients whereas 1 patient had arterial hypertension. Analysis of the lipid profile showed following results: TC 195.33 ± 53.31 mg/dl, HDL 67.55 ± 13.65 mg/dl, LDL 113.60 ± 43.92 mg/dl, and TG 94.53 ± 41.64 mg/dl. None of subjects had cancer disease or were treated with corticosteroids before.

Most frequently occurring comorbidities were CVD (76% of patients), from which over 55% were the arterial hypertension (AH). The next in the order of occurrence of the accompanying diseases in the researched population were autoimmune diseases (such as: Hashimoto disease, antiphospholipid syndrome, psoriasis, or sclerosis multiplex) (34.21%), ophthalmic (31.58%), rheumatologic and dyslipidemia (both 26.31%), allergies (23.68%), neurological (13.16%), diabetes (10.53%), and respiratory system (7.89%).

The analysis of therapy in the SLE group was as follows: 44.74% of the study population were treated with corticosteroids (dose <7.50 mg/day), and the same percentage of patients used chloroquine. Other immunosuppressants, such as azathioprine or methotrexate, were used by, respectively: 10.52% and 2% of patients, whereas 10.52% of patients were treated with combination of corticosteroids and antimalarial drugs. Five patients (13%) did not take drugs against lupus.

Among the characteristic symptoms of SLE, occurring with the highest frequency was pain in at least two joints (84.21%), followed by skin hypersensitivity to light (81.58%), and the third most common symptom was erythema in the shape of a butterfly on the face (78.95%). The next most common symptoms were erythema elsewhere (65.79%), weakness (63.16%), headache (60.53%), dry eye (47.37%), visual impairment (34.21%), and Raynaud's phenomenon (26.31%). There were less frequent symptoms such as depression, other nervous system disorders and oral ulcers (each parameter, 23.68%), and anaemia (18.42%). Orientation (15.79%), memory (13.15%), and speech (10.53%) disorders were even less frequent in the study population. Epileptic seizures occurred with the lowest frequency of all the symptoms (2.63%). SLE characteristics, serological data, and treatment used are included in Table 2.

Abnormal body weight (BMI >25 kg/m^2^) was more frequent in the SLE patients' group, but the difference was not statistically significant between groups (55.26% vs. 52.38%, *p* = 0.6159). LVEF values were significantly lower in patients with SLE compared to the control group (53.92% ± 6.46 vs. 58.67% ± 4.69, *p* = 0.0044). The thickness of the IMT complex was higher in the study population, both in the left (1.22 mm ± 0.27 mm vs. 0.70 mm ± 0.21 mm, *p* = 0.0001) and right common carotid artery (1.16 mm ± 0.26 mm vs. 0.59 mm ± 0.15 mm, *p* = 0.0001). IMT values did not differ statistically between patients who smoked cigarettes and those without addiction (in the left and right carotid artery, respectively, 1.29 mm ± 0.17 mm vs. 1.20 mm ± 0.28 mm, *p* > 0.2071, and 1.27 mm ± 0.19 mm vs. 1.13 mm ± 0.27 mm, *p* = 0.3275).

There was weak positive correlation strength between the duration of the disease and the left IMT (0.27) and right carotid artery (0.10). The weak negative correlation occurred between BMI and LVEF parameters, while compared to the duration of the disease (respectively, 0.13 and −0.19). Tested parameters are included in Table 3.

**Figure 1:**
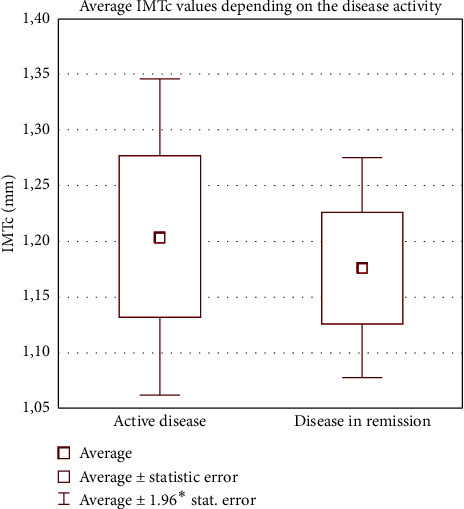
Average IMT values depending to the disease activity in the study group. IMT sin: sinistram carotid intima-media complex thickness; IMT dex: dextram carotid intima-media complex thickness. Source: own studies.

Analysis of the parameters depending on the disease activity according to the SLEDAI-2K showed that patients with active SLE (≥6 points) had significantly lower LVEF values comparing to patients with lower scoring (51.82% ± 7.31% vs. 56.09% ± 5.18%, *p* = 0.0475). The average IMT and BMI were lower in the group with active disease, and the difference was not statistically significant (respectively, 1.17 ± 0.23 vs. 1.20 ± 0.29, *p* = 0.8949, and 27.15 ± 5.12 vs. 25.94 ± 4.06, *p* = 0.5571). QRISK®3CVS risk score showed higher values among patients with active disease, but the difference was not relevant. Among these particular groups, 10 (26.31%) patients with ≥6 points were treated with GCS, comparing to 7 (18.42%) without active disease. Similarly, the lipid profile analysis for SLEDAI-2K showed no significant differences between those groups. Results achieved are presented in Table 4, and the IMT values are presented in Figure 1.

In the matter of treatment with corticosteroids, patients on GCS showed significantly higher QRISK®3 score (respectively, 20.73 ± 15.63 vs. 11.73 ± 9.70, *p* = 0.0267), total cholesterol (226.62 ± 49.76 vs. 187.18 ± 38.47, *p* = 0.0272), and HDL (72.75 ± 16.58 vs. 57.72 ± 18.09, *p* = 0.0238). The values of LVEF (54.47 ± 6.25 vs. 53.95 ± 6.83, *p* = 0.9766), average IMT (1.21 ± 0.25 vs. 1.17 ± 0.24, *p* = 0.7802), and BMI (27.11 ± 5.55 vs. 26.21 ± 3.81, *p* = 0.9532) were higher among the group treated with corticosteroids, but none of these differences revealed a statistical significance. Results obtained from the analysis are presented in Table 5.

## 4. Discussion

SLE is a rare disease in Polish geographical location, and its incidence is estimated around 52 cases/100 000 people [14]. It should be considered as a drawback for large research studies since it is objectively hard to gather a large study group. On average, the risk of SLE is up to 8–15 times higher for women than for men (among adults, and for children, it is 2–8 : 1). One of the main determinants of the SLE is age, because the beginning of the disease is most often recorded in reproductive age. However, this does not mean that the occurrence of the SLE in children is rare, because it accounts for up to 20% of all cases of disease [15]. In these conditions, it is extremely hard to gather a large study group in one centre which is a limitation in the presence of often divergent data.

The SLE is found to be a risk factor for cardiovascular death, and some sources provide information that this mentioned population incidence of myocardial infarction occurrence is 50 times higher than in healthy adults [16]. As an inflammatory disease, it is linked to elevated production of molecules such as NF-KB, TNF-alpha, or IL-1. These particles have proven negative effects on the endothelium, which potentially marks one of the grounds that can contribute to development of the CVD [17,18]. Furthermore, SLE presence is linked to raised levels of atherogenic molecules, such as homocysteine and leptin [19]. However, not only is it the disease that contributes to the atherosclerosis development, but also a vast role is being played by the pharmacotherapy directed to the SLE. One of the therapeutic lines in the treatment is a long-term corticosteroids usage which due to their mechanism of action may lead to increased glycaemia and lipid metabolism disorder. It has not been clarified, however, if in the SLE therapy using corticosteroids significantly contributes to faster occurrence of the risk factors, or if the effects of inhibiting disease process are more beneficial in protection against CVD [20].

All of these information leads to fact that not only is atherosclerosis, present among SLE patients, linked to the traditional risk factors, such as age, sex, diet, or tobacco, but also it is conditioned by CMD and disease-specific factors and by pharmacotherapy against SLE [5,21].

Increased thickness of the intima-media complex in common carotid arteries is also linked with atherogenesis and smooth muscle cell growth, among SLE patient's measurements of this parameter is beneficial due to two facts. Firstly, it allows to diagnose atherosclerosis earlier, and secondly, it is a remarkable method to find patients with increased cardiovascular risk. This could be a new way for fast therapeutic respond and avoidance of complications, due to fact that patients with SLE have significantly increased IMT values when compared to the healthy controls. This can indicate that in SLE risk of developing atherosclerosis, cardiovascular-related death is greater [22]. Reference range for IMT indicates values up to 0.9 mm as a normal, and values greater than 1.5 mm could give a suspicion of the presence of atherosclerotic plaque, which is a strong cardiovascular risk factor [7–9]. There are other studies in which significantly higher values of IMT have been confirmed in patients with SLE when compared to control groups [6,7,21].

In the research paper, its authors had taken into account various cardiovascular risk factors accompanying patients with SLE, the effect of immunosuppressive treatment on their values, and correlations between them [4]. The study group consisted of 103 patients with SLE. Presented results showed that IMT was significantly thicker in the SLE group while compared to healthy controls (*p* = 0.0001), and that there was a weak positive correlation of the IMT with the disease duration (*r* = 0.30). In the conducted study, results coincide with mentioned above, both in the aspect of lack of strong correlation, and in the difference of IMT values (respectively, IMT in the left (*p* = 0.00015, *r* = 0.27) and right (*p* = 0.0001, *r* = 0.10) CCA).

On the other hand, the study which counted 392 adults with SLE was conducted in order to assess the relationship between cardiovascular events (i.e., ischemic stroke myocardial infarction) and the value of IMT in adult women with SLE [23]. For this purpose, the frequency of these incidents was compared with the presence of carotid artery plaques and the IMT. It was shown that in patients with higher values of these parameters, cardiovascular events (*p* < 0.01) were statistically more frequent and the presence of atherosclerotic plaque was a strong marker of their occurrence. In the material collected from this study, IMT values are significantly higher in the patient population (*p* = 0.0001 in the left and *p* = 0.0001 in the right carotid artery).

When it comes to the assessment of the heart structure and functioning in the SLE group, TTE is the method of choice. One of the most significant parameters measured is the LVEF, which could correlate with the disease severity. Its reduction could indicate an inflammation in the heart, caused by the SLE antibodies. To confirm this hypothesis, next to the magnetic resonance, one can take a fragment of the heart muscle for histopathological examination, during an endomyocardial biopsy, which is already an invasive diagnosis. Myocarditis is a complication that causes dysfunction of the endothelium in the small blood vessels, which accelerates atherogenesis [11]. However, LVEF is primarily a marker of heart failure, and values below 50% may indicate a significant disorder of left ventricular systolic function. The development of insufficiency is an unfavourable prognostic factor, increasing the risk of sudden cardiac death, which in the case of SLE patients further complicates the therapeutic process. It is proven that in SLE cases, the LVEF values are statistically lower than normal [2,3]. The study of Zhang is important in the context of assessment of myocardial functioning in patients with SLE. Its main goal was to determine the risk factors for the occurrence of lupus myocarditis, and to compare echocardiographic parameters in this group of patients [24]. In the results presented by the researchers, it was found that in 92% of patients, LVEF values were below the norm (LVEF <50%) and that in patients with initially low LVEF, despite intensive immunosuppressive therapy, there was no improvement. The reduced left ventricular systolic function is therefore an unfavourable prognostic factor for the patient.

In the research, we have found that heart structure differs in SLE group, the left ventricular end-diastolic diameter was significantly higher than in the control group (45.60 ± 6.72 vs. 40.75 ± 6.83 mm, *p* = 0.0247), and the LVEF on average was lower in that group (50.82 ± 7.01% vs. 57.68 ± 4.56%, *p* = 0.0001) [25]. The SLE also had an impact on everyday lives, as study showed that during the 6-minute walk test, SLE patients covered much less distance than healthy adults (547.86 ± 87.59 vs. 595.37 ± 78.56 m, *p* = 0.0472) and that activity of the disease was an important factor, as patients in regression achieved better results than patients with active SLE, according to the SLEDAI-2K (505.57 ± 75.55 vs. 582.35 ± 71.84).

Additionally, in the study, researchers focused on the assessment of myocardial dysfunction among 80 patients with SLE, dividing them into a population with positive and negative anticardiolipin antibodies [26]. The results presented showed that patients with SLE have lower LVEF values compared to a healthy control group and that there are no difference between the occurrence of anticardiolipin antibodies and the LVEF. Comparing that to results of this study, the LVEF values were also lower, but the difference was statistically significant (*p* = 0.0044) between groups.

Due to the causes which were mentioned earlier, the lipid profile of SLE patients could differ from normal one. It is common for this group to have dyslipidemia, which could be partially caused by the disease itself, as well as by the treatment process, and the use of medications could easily alter the lipid profile [27]. For example, the corticosteroids are linked to prevalence of the dyslipidemia, and they are increasing risk of a diabetes mellitus type 2, but on the other side, they may have an impact on a carotid plaque [28].

The QRISK®3 cardiovascular event risk calculator was designed as a successor of a second version. The newest one contains additional variables, counting rheumatoid diseases such as SLE that could affect the outcome. It had been validated on large cohorts in the United Kingdom and shown a better performance in estimating a cardiovascular risk than the Anderson Framingham equation [13,29,30]. It is important to bear in mind that this risk scale was designed for the UK population, which may differ in particular ways from Polish patients. In the research, which was published in 2018 in Lupus Journal, authors found that QRISK®3 was significantly more reliable in capturing SLE patients with increased CVS risk than older version-QIRKS®2 (29 patients vs. 8 patients, *p* = 0.0001) and from Framingham equations (29 vs. 5 patients, *p* = 0.0001). However, a small study group in mentioned article requires further research [31].

## 5. Conclusions

The research proves that patients suffering from SLE are particularly predisposed to the onset of atherosclerotic plaque in the carotid arteries, which promotes the risk of cardiovascular events as the disease progresses. The patients with SLE from researched group showed a significantly decreased LVEF, which may in the future correlate with the occurrence of heart failure. It is advisable that the SLE patient group should be under constant cardiac control due to the significant risk of CVD. Special attention should be paid to the autoimmune component, which may have a significant impact on the pathogenesis of atherosclerotic lesions. The activity of the disease according to the SLEDAI-2K scale may have an impact on the LVEF values which was significantly decreased in the group with active disease, but further thorough investigation is required to fully evaluate the impact of individual components of the disease and its treatment on the cardiovascular disease development and mortality. The patient with corticosteroids treatment showed an increased QRISK®3 score, which indicates a higher cardiovascular risk than patients on other medications, and similar situation occurred among total cholesterol and HDL fraction.

## Figures and Tables

**Table 1:** Anthropometric characteristics, SLEDAI-2K, and lipid profile of control and study groups.

Patient characteristics	Study group	Control group	*p* value
Age (years)	56.45 ± 11.01	51.91 ± 12.38	0.3212
Female (%) (*n*)	89.94 (34)	90.47 (19)	0.8122
Height (cm)	161.59 ± 18.44	163.60 ± 6.84	0.6757
Weight (kg)	72.05 ± 16.01	73.09 ± 16.43	0.7235
BMI (kg/m^2^)	26.61 ± 4.59	27.26 ± 4.71	0.6159
Smokers (%) (*n*)	18.42 (7)	4 (1)	0.3174
SLEDAI-2K	6.89 ± 5.51	—	—
SLEDAI-2K >6	44.74 (17)	—	—
Disease duration (years)	10.21 ± 7.69	—	—
Time of treatment (years)	10.21 ± 7.69	—	—
Lipid profile			
TC (mg/dl)	205.74 ± 47.83	195.33 ± 53.31	0.4622
HDL (mg/dl)	64.55 ± 18.76	67.55 ± 13.65	0.8122
LDL (mg/dl)	118.41 ± 43.62	113.60 ± 43.92	0.6898
TG (mg/dl)	114.09 ± 35.89	94.53 ± 41.63	0.1806

BMI : body mass index; SLEDAI-2K : Systemic Lupus Erythematosus Disease Activity Index; TC : total cholesterol; HDL : high-density cholesterol; LDL : low-density cholesterol; TG : triglycerides. Source: own studies.

**Table 2:** Treatment, SLE specific symptoms, serological data, and treatment used in the SLE group.

Treatment	Study group *n* (%)
Corticosteroids	17 (44.74)
Azatioprine	4 (10.52)
Chloroquine	17 (44.74)
Chloroquine with metotrexate	1 (2.63)
GCS with chloroquine	4 (10.52)
GCS with azatioprine	2 (5.26)
Without treatment	6 (15.79)
SLE characteristics	
Facial erythema (“butterfly rash”)	30 (78.95)
Erythema elsewhere	25 (65.79)
Skin hypersensitivity to light	31 (81.58)
Oral ulcers	9 (23.68)
Synovitis (>2 joints)	32 (84.21)
Nonscarring alopecia	20 (52.63)
Raynaud symptom	10 (26.31)
Serological	
ANAs (+)	34 (89.47)
dsDNA	20 (52.63)
RNP	2 (5.26)
SSA/Ro	19 (50.00)
SSB/La	3 (7.89)
Sm	7 (18.42)

GCS: corticosteroids; ANAs : antinuclear antibodies; dsDNA : anti-double-strand DNA antibodies; RNP : antiribonucleoprotein antibodies; SSA/Ro : anti-Ro antibodies; SSB/La : anti-La antibodies; Sm : anti-Smith antibodies. Source: own studies.

**Table 3:** Results of studied parameters (LVEF, IMT, and BMI) in the control and study groups.

Studied parameters	Study group	Control group	*p* value	*r*
LVEF (%)	53.92 ± 6.46	58.67 ± 4.69	**0.0044**	0.27
IMT sin (mm)	1.22 ± 0.27	0.7 ± 0.21	**0.0001**	0.10
IMT dex (mm)	1.16 ± 0.26	0.59 ± 0.15	**0.0001**	0.13
BMI (kg/m^2^)	26.61 ± 4.59	27.26 ± 4.71	0.6159	−0.19

LVEF : left ventricular ejection fraction; IMT sin: sinistram carotid intima-media complex thickness; IMT dex: dextram carotid intima-media complex thickness; BMI : body mass index. Source: own studies.

**Table 4:** Comparison of disease activity according to the SLEDAI-2K in the study group.

Comparison with disease activity	SLEDAI <6	SLEDAI ≥6	*p* value
LVEF (%)	56.09 ± 5.18	51.82 ± 7.31	**0.0475**
IMT AVG (mm)	1.17 ± 0.23	1.20 ± 0.29	0.8949
BMI (kg/m^2^)	27.15 ± 5.12	25.94 ± 4.06	0.5571
QRISK®3 (%)	14.57 ± 9.25	17.22 ± 17.20	0.7027
Coriticosteroids	7 (18.42%)	10 (26.31%)	0.6913
TC (mg/dl)	204.76 ± 45.96	206.84 ± 51.34	0.6168
HDL (mg/dl)	65.02 ± 18.17	63.98 ± 20.07	0.9136
LDL (mg/dl)	116.84 ± 47.87	120.16 ± 39.78	0.4687
TG (mg/dl)	117.56 ± 28.61	117.56 ± 41.61	0.6643

LVEF : left ventricular ejection fraction; IMT AVG : carotid intima-media complex thickness average value; BMI : body mass index. QRISK®3: cardiovascular event risk calculator; TC : total cholesterol; HDL: high-density cholesterol; LDL: low-density cholesterol; TG: triglycerides. Source: own studies.

**Table 5:** Comparison between corticosteroids usage and other therapeutic strategies in the study group.

Comparison with corticosteroids usage	Other drugs	GCS used (<7.50 mg/day)	*p* value
LVEF (%)	53.95 ± 6.83	54.47 ± 6.25	0.9766
IMT AVG (mm)	1.17 ± 0.24	1.21 ± 0.25	0.7802
BMI (kg/m^2^)	26.21 ± 3.81	27.11 ± 5.55	0.9532
SLEDAI-2K	7.43 ± 6.10	6.00 ± 4.62	0.6281
QRISK®3 (%)	**11.73** **±** **9.70**	**20.73** **±** **15.63**	**0.0267**
TC (mg/dl)	**187.18** **±** **38.47**	**226.62** **±** **49.76**	**0.0272**
HDL (mg/dl)	**57.72** **±** **18.09**	**72.75** **±** **16.58**	**0.0238**
LDL (mg/dl)	114.45 ± 50.93	122.85 ± 34.74	0.3789
TG (mg/dl)	115.79 ± 37.87	112.04 ± 34.56	0.8149

LVEF : left ventricular ejection fraction; IMT AVG : carotid intima-media complex thickness average values, BMI : body mass index; SLEDAI-2K : Systemic Lupus Erythematosus Disease Activity Index; QRISK®3: cardiovascular event risk calculator; TC : total cholesterol; HDL: high-density cholesterol; LDL: low-density cholesterol; TG: triglycerides. Source: own studies.

## Data Availability

The data used to support the findings of this study are included within the article and have also been deposited in the PubMed database and the ClinicalKey repository.

## References

[1] Fortuna G., Brennan M. T. (2013). Systemic lupus erythematosus. *Dental Clinics of North America*.

[2] Doria A., Iaccarino L., Sarzi-Puttini P., Atzeni F., Turriel M., Petri M. (2005). Cardiac involvement in systemic lupus erythematosus. *Lupus*.

[3] Chen J., Tang Y., Zhu M., Xu A. (2016). Heart involvement in systemic lupus erythematosus: a systemic review and meta-analysis. *Clinical Rheumatology*.

[4] Kisiel B., Kruszewski R. A., Raczkiewicz A. (2015). Systemic lupus erythematosus: the influence of disease-related and classical risk factors on intima media thickness and prevalence of atherosclerotic plaques-a preliminary report. Beneficial effect of immunosuppressive treatment on carotid intima media thickness. *Acta Cardiologica*.

[5] Severino P., D’Amato A., Pucci M. (2020). Ischemic heart disease and heart failure: role of coronary ion channels. *International Journal of Molecular Sciences*.

[6] Luijendijk P., Lu H., Heynneman F. B. (2014). Increased carotid intima-media thickness predicts cardiovascular events in aortic coarctation. *International Journal of Cardiology*.

[7] Simova I. (2015). Intima-media thickness: appropriate evaluation and proper measurement, described. *E-journal of the ESC Council for Cardiology Practice*.

[8] O’Leary D. H., Polak J. F., Kronmal R. A. (1999). Carotid-artery intima and media thickness as a riskfactor for myocardialinfarction andstroke in older adults. *New England Journal of Medicine*.

[9] Chambless L. E., Heiss G., Folsom A. R., Szklo M., Sharrett A. R., Clegg L. X. (1997). Association of coronary heart disease incidence with carotid arterial wall thickness and major risk factors: the atherosclerosis risk in communities (ARIC) study, 1987-1993. *American Journal of Epidemiology*.

[10] Stein J. H., Korcarz C. E., Hurst R. T. (2008). Use of carotid ultrasound to identify subclinical vascular disease and evaluate cardiovascular disease risk: a consensus statement from the American society of echocardiography carotid intima-media thickness task force endorsed by the society for vascular medicine. *Journal of the American Society of Echocardiography*.

[11] Mach F., Baigent C., Catapano A. L. (2020). 2019 ESC/EAS Guidelines for the management of dyslipidaemias: lipid modification to reduce cardiovascular risk: The Task Force for the management of dyslipidaemias of the European Society of Cardiology (ESC) and European Atherosclerosis Society (EAS). *European Heart Journal*.

[12] Perel-Winkler A., Bokhari S., Perez-Recio T., Zartoshti A., Askanase A., Geraldino-Pardilla L. (2018). Myocarditis in systemic lupus erythematosus diagnosed by 18F-fluorodeoxyglucose positron emission tomography. *Lupus Science and Medicine*.

[13] Hippisley-Cox J., Coupland C., Vinogradova Y., Robson J., Brindle P. (2008). Performance of the QRISK cardiovascular risk prediction algorithm in an independent UK sample of patients from general practice: a validation study. *Heart*.

[14] Śliwczyński A., Brzozowska M., Iltchev P. (2015). Changes in the morbidity and costs of systemic lupus erythematosus in Poland in the years 2008–2012. *Reumatologia*.

[15] Pons-Estel G. J., Ugarte-Gil M. F., Alarcón G. S. (2017). Epidemiology of systemic lupus erythematosus. *Expert Review of Clinical Immunology*.

[16] Mauro D., Nerviani A. (2018). Endothelial dysfunction in systemic lupus erythematosus: pathogenesis, assessment and therapeutic opportunities. *Reviews on Recent Clinical Trials*.

[17] Petri M. (2000). Detection of coronary artery disease and the role of traditional risk factors in the hopkins lupus cohort. *Lupus*.

[18] Bruce I. N., Gladman D. D., Urowitz M. B. (2000). Premature atherosclerosis in systemic lupus erythematosus. *Rheumatic Disease Clinics of North America*.

[19] Khairy N., Ezzat Y., Naeem N., Taha R., Wesam R. (2017). Atherosclerosis biomarkers in female systemic lupus erythematosus patients with and without cardiovascular diseases. *The Egyptian Rheumatologist*.

[20] McMahon M., Hahn B. H., Skaggs B. J. (2011). Systemic lupus erythematosus and cardiovascular disease: prediction and potential for therapeutic intervention. *Expert Review of Clinical Immunology*.

[21] Zeller C., Appenzeller S. (2008). Cardiovascular disease in systemic lupus erythematosus: the role of traditional and lupus related risk factors. *Current Cardiology Reviews*.

[22] Wu G.-C., Liu H.-R., Leng R.-X. (2016). Subclinical atherosclerosis in patients with systemic lupus erythematosus: a systemic review and meta-analysis. *Autoimmunity Reviews*.

[23] Kao A. H., Lertratanakul A., Elliott J. R. (2013). Relation of carotid intima-media thickness and plaque with incident cardiovascular events in women with systemic lupus erythematosus. *The American Journal of Cardiology*.

[24] Zhang L., Zhu Y.-L., Li M.-T. (2015). Lupus myocarditis. *Chinese Medical Journal*.

[25] Przywara-Chowaniec B., Dyrcz D., Bereś M., Harpula J., Nowak A., Tomasik A. (2019). Echocardiography and 6-minute walk test for assessing disease progression in systemic lupus erythematosus. *Cardiology and Cardiovascular Medicine*.

[26] Barutcu A., Aksu F., Ozcelik F. (2015). Evaluation of early cardiac dysfunction in patients with systemic lupus erythematosus with or without anticardiolipin antibodies. *Lupus*.

[27] Ardoin S. P., Sandborg C., Schanberg L. E. (2007). Review: management of dyslipidemia in children and adolescents with systemic lupus erythematosus. *Lupus*.

[28] Roman M. J., Shanker B.-A., Davis A. M. D. (2003). Prevalence and correlates of accelerated atherosclerosis in systemic lupus erythematosus. *New England Journal of Medicine*.

[29] Hippisley-Cox J., Coupland C., Vinogradova Y., Robson J., May M., Brindle P. (2007). Derivation and validation of QRISK, a new cardiovascular disease risk score for the United Kingdom: prospective open cohort study. *BMJ*.

[30] Collins G. S., Altman D. G. (2009). An independent external validation and evaluation of QRISK cardiovascular risk prediction: a prospective open cohort study. *BMJ*.

[31] Edwards N., Langford-Smith A., Parker B. J. (2018). QRISK3 improves detection of cardiovascular disease risk in patients with systemic lupus erythematosus. *Lupus Science and Medicine*.

